# Supplement Type Impact on the Performance and Nutrient Dynamics of Nursing Does and Kids Raised in Woodlands

**DOI:** 10.3390/ani14010068

**Published:** 2023-12-23

**Authors:** Bhuwan Shrestha, Uma Karki, Santoshi Chaudhary, Anand Tiwari, Lila B. Karki

**Affiliations:** 1 College of Agriculture, Environment and Nutrition Sciences, Tuskegee University, Tuskegee, AL 36088, USA; ukarki@tuskegee.edu (U.K.); smc7847@psu.edu (S.C.); atiwari3@umd.edu (A.T.); 2 Department of Animal Science, Texas A&M University, College Station, TX 77843, USA; 3 Department of Veterinary and Biomedical Sciences, Pennsylvania State University, State College, PA 16802, USA; 4 Department of Animal and Avian Sciences, University of Maryland, College Park, MD 20742, USA; 5 Department of Agriculture, Food and Resource Sciences, University of Maryland Eastern Shore, Princess Anne, MD 21853, USA; lkarki@umes.edu

**Keywords:** corn, fecal N, hay, Kiko, silvopasture, supplemental grazing

## Abstract

**Simple Summary:**

The mature Kiko wethers performed satisfactorily in woodlands, whereas the performance of young and growing animals was suboptimal, and they required supplementation. However, information is scant on supplementation strategies for nursing does and their kids stocked in woodlands. This study aimed to evaluate the supplementation strategies and assess the associated performance of nursing Kiko does and kids and the nutritional status of does as reflected in their feces when stocked in woodlands. Two supplementation strategies were evaluated: (1) supplemental grazing in adjacent silvopasture plots for 3–4 h/day and (2) supplementation with corn at the rate of 0.5% of their metabolic body weight and free-choice hay. Nursing Kiko does raised in woodlands with supplemental grazing showed a better FAMACHA score and browsing activities at a greater height compared to their counterpart does supplemented with hay and corn. The fecal nitrogen concentration was higher in does with supplemental grazing compared to those supplemented with hay and corn. The supplemental grazing was found to be a better option than supplementing with hay and corn for nursing Kiko does raised in woodlands.

**Abstract:**

The influence of different supplement types on the performance and nutrient dynamics of goats stocked in woodlands is not known. The objective of this study was to evaluate the effect of supplement type on the performance and the concentration of fecal nutrients of nursing does and the performance of kids raised in woodlands. One group of goats (SG, 9 does; 18 kids) was allowed supplemental grazing in adjacent silvopasture plots for 3–4 h daily and another group (SF, 8 does; 15 kids) was supplemented with corn (0.5% of metabolic weight) and ad libitum hay. Vegetation samples were collected and analyzed for productivity and quality (crude protein, CP; acid detergent fiber, ADF; neutral detergent fiber, NDF). The quality of the hay (N, ADF, NDF) and fecal samples (N, P, Ca) was analyzed. The animals’ live weight, FAMACHA score, and body condition score were collected. The browsing height for does consuming woodland vegetation was measured. Data were analyzed using the GLM procedure, Mixed procedure, and MEANS procedure in SAS 9.4. SG does showed better FAMACHA scores vs. SF does (*p* < 0.05). Fecal N and ADF were greater (*p* < 0.0001) in SG does vs. SF does. The findings showed a better performance and greater concentration of fecal nutrients in SG does vs. SF does, suggesting grazing quality pastures is a better option than using feedstuffs to supplement nursing does in woodland.

## 1. Introduction

Goats are raised under different rearing systems such as extensive [1], semi-intensive, and intensive systems [2]. In the southeast US, goats are mainly raised extensively on pastures dominated by seasonal grasses, which have poor productivity and are not abundant enough to feed small ruminants year-round. Forage remains deficient for the animals’ requirements from September/October to March/April when winter forages are not grown [1]. Extending the grazing opportunities for small ruminants could complement year-round grazing, for which woodland integration can be one of the viable options. Woodland grazing allows the use of forage produced in pastures for hay making and stockpiling, which can be used to feed animals during the period of lean forage growth. Different understory vegetations in woodlands provide a high number of nutrients to the animals. Numerous woody species in the woodland understory contain condensed tannins, the plants’ secondary metabolites, which function as an anthelmintic [1,3]. Additionally, animals in woodlands have good access to browse, which inhibits the interaction with gastrointestinal (GI) parasites that reside on and close to the ground surface. Therefore, woodland grazing should be helpful in minimizing the GI parasite infestation in animals in addition to fulfilling their nutrient needs [1,3].

The United States has an enormous land area occupied by woodlands (33%; 310 million hectares), which largely contributes to the economic and environmental benefits. In the southeastern United States, most of the land area (62%) is covered by woodlands [4]. Alabama has 69% of its total land occupied by woodlands [5]. As there are numerous plant species that are readily consumed by small ruminants present in the woodland understory [6,7,8], there are excellent prospects for grazing goats in woodlands for multiple benefits. One of the major benefits of grazing is the biological control of the woodland understory and reducing costs that would otherwise be incurred for various forest management practices like mechanical thinning, prescribed burning, and herbicide application to control the understory vegetation [2].

The cost associated with conventional methods for controlling woodland understory vegetation varies from USD 77.8/ha for prescribed burning to USD 399.4/ha for the mechanical method [9]. Goat grazing can be a viable alternative to conventional methods for managing understory woodland vegetation. A woodland grazing study by Khatri (2016) [6] found that young Kiko goats utilized 50–75% of the understory vegetation available in woodlands up to the height of 1.5 m from the ground surface. Bhattarai (2019) [7] reported comparable results with mature Kiko wethers and Katahdin rams as they were found to consume the understory vegetation available up to the average heights of 1.6 m and 1.1 m, respectively. Nonetheless, the performance of goats in woodlands may vary based on their age and physiological stage. Previous woodland studies by Bhattarai (2019) [7] reported a satisfactory performance of mature Kiko wethers and Katahdin rams. However, the performance of young and growing goats was suboptimal, and they required supplementation [6,10]. As the need for nutrients is high during the lactation period, the stocking of lactating animals in woodlands without any supplement could be challenging. The demand for nutrients increases during the early to mid-lactation period and slightly decreases in late lactation. The NRC (2007) [11] recommendation of energy for non-dairy goats weighing 40 kg at early lactation is 11.88 MJ of ME to raise a single kid and 15.77 MJ of ME to raise twins. For goats weighing 40 kg at late lactation, 8.66 MJ of ME is recommended for does nursing a single kid and 10.13 MJ of ME for does nursing twins [11]. Animals will be vulnerable to diseases and parasites if they consume insufficient nutrition during late gestation, parturition, and early lactation. This is because inadequate nutrition weakens the immune functions, which directly affects the performance of animals [12]. Therefore, lactating animals require proper supplementation when stocked in woodlands for satisfactory performance. However, not much is known about how different supplement types influence the performance of goats stocked in woodlands.

Previous woodland grazing studies revealed that the better performance of small ruminants was mainly limited by energy [13]. Corn is commonly used as the source of energy to feed farm animals, as it is a good source of energy (TDN—88%; CP—9%) [11]. Walz et al. (2003) [14] reported that supplementing Spanish kids in pens with corn (190 g/day/kid) and ad libitum coastal bermudagrass (*Cynodon dactylon* (L.) Pers.) hay increased the DM intake by 22% (*p* < 0.01) and daily body weight gain by 24.5 g (*p* < 0.01) vs. non-supplemented Spanish kids. Corn supplementation (0.5% of body weight) for Spanish × Boer wethers grazing in native rangelands increased the average daily gain by 61% vs. non-supplemented wethers. Similarly, corn supplementation (1% of body weight) for Spanish × Boer wethers grazing in improved (cultivated) pastures with annual legumes increased their average daily weight gain by 31% vs. non-supplemented wethers [15]. Other than supplementing with feedstuffs animals grazing in woodlands, quality pastures could be used as supplements [2]. However, studies have not been conducted to determine the effectiveness of using pastures for supplementing animals stocked in woodlands.

Nutrients such as nitrogen (N), phosphorus (P), and calcium (Ca) excreted in feces can be assessed and used for predicting the digestibility and energy content in the animal’s diet [16,17]. Nonetheless, studies to evaluate the nutritional status of goats from their feces when stocked in woodlands are limited. The study tested the hypothesis that nursing does and kids stocked in woodlands would perform equally well, and does would have similar fecal nutrients with either supplemental grazing or feedstuffs. The objective of the study was to evaluate the effect of supplement type on the performance and the concentration of fecal nutrients of nursing does and the performance of kids raised in woodlands.

## 2. Materials and Methods

### 2.1. Study Site

**Figure 1:**
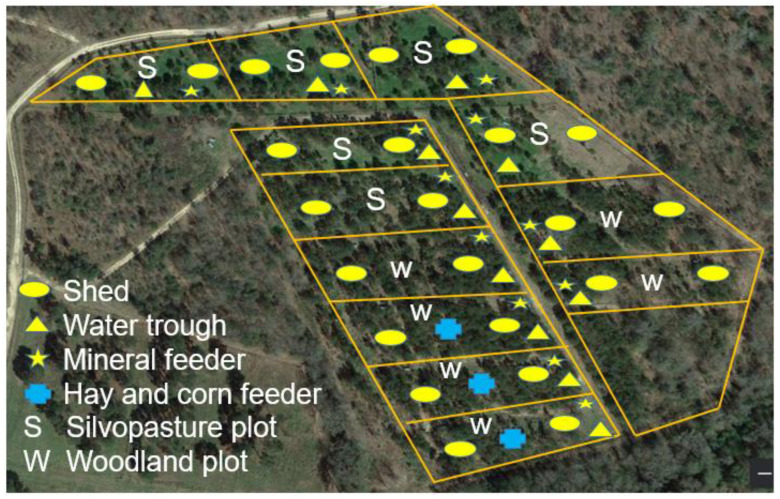
Diagram of the study site showing the locations of shelters, water troughs, mineral feeders, hay and grain feeders in study plots.

The study was conducted at the Atkins Agroforestry Research and Demonstration Site, Tuskegee University, Tuskegee, Alabama, USA (Latitude 32°26′34.0″ N, Longitude 85°43′57.4″ W) from July to October 2021 (89 days). The study site consisted of Cowarts loamy sand (60%; slope 5–15%) and Uchee loamy sand (40%; slope 1–5%) [18]. The site had a humid subtropical climate and an average annual rainfall of 140 cm. The average maximum and minimum temperatures during the study period were 31 °C and 22 °C, respectively, with an average rainfall of 43 cm. The average maximum and minimum relative humidity levels were 94% and 55%, respectively. These values were calculated from the secondary weather data retrieved from www.wunderground.com recorded at the Montgomery regional airport station for Tuskegee Institute, AL. This station was located approximately 72 km west of the study site. The site consisted of six woodland plots and six adjacent silvopasture plots (Figure 1). Each woodland plot contained 16-year-old mixed southern pines (longleaf (*Pinus palustris* Mill.) and loblolly (*Pinus taeda* L.)) and various understory plant species (Table 1). Silvopasture plots contained mixed southern pines (longleaf and loblolly) with planted forages—bahiagrass (*Paspalum notatum* Flueggé), cowpeas (*Vigna unguiculata* (L.) Walp.), sericea lespedeza (*Lespedeza cuneata* (Dum. Cours.) G. Don), and crabgrass (*Digitaria radicosa* (J. Presl) Miq.). Each plot (0.4 ha) was fenced on all four sides and consisted of a watering system, mineral feeders (1 or 2), and portable shelters (2; Port-A-Hut).

### 2.2. Determination of Vegetation Biomass and Quality, and Hay Quality

Vegetation samples were collected from both woodland and silvopasture plots one day prior to each rotational stocking of each plot throughout the study period. Ten representative samples of vegetation per plot were collected randomly by using 1 m^2^ quadrats up to the height of 1.8 m in woodland plots, whereas 0.25 m^2^ quadrats were used to collect samples from silvopasture plots [19]. Fifty percent of the available vegetation within the 1 m^2^ quadrat in woodland plots and all vegetation present within the 0.25 m^2^ quadrats in silvopasture plots were cut 10.2 cm from the ground surface and put individually in pre-weighed paper bags labeled with the date, sample number, and plot number. The collected samples were oven-dried for 72 h at 60 °C [19]. After drying, samples were allowed to cool down for 15–20 min at room temperature and weighed for biomass determination. Dried samples were then ground for quality analyses: crude protein (CP), acid detergent fiber (ADF), and neutral detergent fiber (NDF) using Near-Infrared Spectroscopy (NIRS) in the Agroforestry and Grazing-Land Ecology Lab of Tuskegee University. Total digestible nutrients (TDNs) were derived from NDF values TDN% = (105.2 − 0.667 × NDF) × 0.88. The calibration and validation of the NIRS equation were performed before developing the final NIRS model that was used for spectral reading of the vegetation samples. For calibration, NIRS spectral values and wet chemistry results of the same set of samples were used to develop mathematical relationships. For the validation of the NIRS calibration equation, other samples (which were not used to develop the model) were used. After generating an NIRS model, ground samples were loaded in a rotating cup at room temperature and immediately scanned with an Antaris II FT-NIR Analyzer, Thermo Fisher Scientific (Waltham, MA, USA). The spectra generated with their values were collected and exported to Excel for statistical analyses.

The samples of hay were collected from each square bale provided to animals and then ground for quality analyses of CP, ADF, and NDF using NIRS in the Agroforestry and Grazing-Land Ecology Lab of Tuskegee University.

### 2.3. Research Animals

Nursing Kiko does (17) aged 26–27 months and their 2–3-month-old kids (33) (male: 15 and female: 18) were used for the study (Table 2). Animals were grazed in silvopasture plots before they were brought to the woodland study plots. Does were divided into two uniform groups based on their live weight, FAMACHA score, and body condition score, and kids were allowed to remain with their mothers. Each group of animals was allocated to a separate set of woodland plots and rotated among those plots throughout the study. One group of animals (SG) (9 does; 18 kids) was allowed supplemental grazing in silvopasture plots for 3–4 h/day, and another group of animals (SF) (8 does; 15 kids) was supplemented with ad libitum coastal bermudagrass hay and whole corn (9% CP) at the rate of 0.5% of the animal’s metabolic weight. Animals were moved to new plots from the initial plots once they utilized 50% of the vegetation available in the initial plots (in both woodland and silvopasture). The utilization and availability of vegetation in the study plots were assessed by daily visual observations and the use of photoplots before and after grazing.

### 2.4. Animal Performance Data

To measure animal performance, three performance variables of live weight, body condition score, and FAMACHA score were collected at the beginning after a five-day adjustment, fortnightly during the study, and at the end of the study. The live weight of animals was measured by using a digital weighing scale installed at the handling facility of the study site. BCS was measured on a five-point scale (1–5, 1—extremely lean, 5—obese) by palpating the muscle and fat cover over three specific areas of the animal body: vertebrae, sternum (breastbone), and ribs [20]. In general, goats with BCS values of 2.5–4 are considered healthy [20]. The FAMACHA score ranges from 1 to 5, and 1 and 2 (red color) indicate non-anemic condition, 3 indicates conditional status, and 4 and 5 (pale color) indicate the anemic condition caused by *Haemonchus contortus*, a blood-sucking parasite in small ruminants. Animals in conditional status require close observations and care as appropriate: they need treatment if they are in poor body condition, poor overall health condition, lactation period, and if 5–10% or more of the herd is found to be anemic. Animals with FAMACHA scores of 4 and 5 require immediate treatment and care. The FAMACHA score of each animal was assessed by comparing the color of the conjunctiva on the lower eyelid of both eyes with the matching color on the FAMACHA card. The live weight, FAMACHA score, and BCS were collected by a single trained person (the first author) throughout the study to avoid possible individual bias.

### 2.5. Measurement of Browsing Height

**Figure 2:**
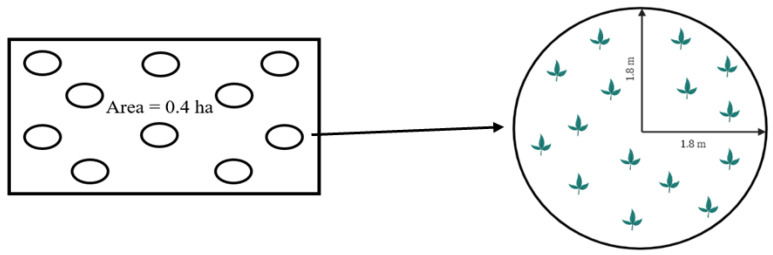
Schematic diagram of observation points (10) in a study plot used to measure the browsing height for goats of woody vegetation.

Ten random observation points per plot were established in woodland plots (Figure 2). The browsing height for goats of woody vegetation was measured on all plants that were present within a radius of 1.8 m from the center of each observation point. The browsing height was measured after each grazing rotation (within one day after the removal of animals from the plot) by using measuring tapes and measuring sticks. The browsing height was measured from the base of the plant to the point up to which animals ate leaves and young twigs.

### 2.6. Fecal Sample Collection and Analysis for Fecal Nutrients

One set of fecal samples was collected from does on Days 1, 33, 60, and 89 of the study by inserting a gloved, lubricated finger into their rectums and extracting fecal pellets. After collection, fecal samples were shipped overnight to the Grazing-Land Animal Nutrition Lab (GANLAB), College Station, TX, USA, maintaining a cold chain for the analysis of fecal nutrients nitrogen and phosphorus. Fecal samples from does were analyzed in the NIR spectrometer in the GANLAB. The major limiting nutrient (energy or protein) in the feedstuffs that does consumed was predicted from the nutrients present in their feces. Additional sets of fecal samples were collected on Days 1, 19, 33, 47, 61, 75, and 89 of the study and analyzed for fecal CP, NDF, and ADF using NIRS in the Agroforestry and Grazing-Land Ecology Lab of Tuskegee University. The TDN content in fecal samples was derived from NDF values [TDN % = (105.2 − 0.667 × NDF) × 0.88].

### 2.7. Data Analyses

All data sets were analyzed in SAS 9.4 by setting the significance level (alpha) for rejecting the null hypothesis at 0.05. Animal performance data (live weight, BCS, and FAMACHA score) were analyzed using the Multivariate Analysis of Variance (MANOVA) option in the Generalized Linear Model (GLM) and repeated measures as performance variables were correlated. The model used to analyze the animal performance data is presented below:

Y1ijY2ijY3ij = µ + αi + (αβ)ij + eij (1)

MANOVA h = Animal group and the interaction of animal group and observation date Repeated factor = Individual animal (2)

where Y (1–3)ij = performance variables of animals from the ith group and jth observation date,

µ = grand mean,

αi = main effect of the group,

(αβ)ij = interaction of the ith group and jth observation date,

eij = error associated with the ith group and jth observation date.

The Mixed Model was used to analyze vegetation biomass and browsing height data with the plot as a random factor. The GLM procedure with the MANOVA option was used to analyze the vegetation quality (CP, NDF, ADF, and TDN) and fecal quality data. The Mixed and GLM models used to analyze vegetation biomass and quality data are presented below.

(i) Mixed Model used for analyzing forage biomass data:

Yi = µ + αi + ei (3)

where Yi = value of an observation for the ith system (silvopasture or woodland),

µ = grand mean,

αi = main effect of the system,

ei = an error associated with the ith system,

Random factor = Research plot.

(ii) Mixed Model used for analyzing the browsing height data:

Yi = µ + αi + ei(4)

where Yi = value of an observation for the ith group (1 or 2),

µ = grand mean,

αi = main effect of the group,

ei = an error associated with the ith group,

Random factor = Research plot.

(iii) Model used for the GLM procedure with the MANOVA option to analyze the forage quality data:

Y1iY2iY3iY4i = µ + αi + ei (5)

MANOVA h = System (6)

where Y (1–4)i = forage quality variables,

µ = grand mean, αi = main effect of the ith system,

ei = error associated with the ith system.

Fecal quality data were analyzed using the GLM procedure with the MANOVA option and repeated measures, and the model is presented below.

Y (1–3)ij = µ + αi + (αβ)ij + eij(7)

MANOVA h = Animal group and the interaction of animal group and observation date Repeated factor = Individual animal(8)

where Y (1–3)ij = fecal nutrient variables for the ith group and jth observation date,

µ = grand mean, αi = main effect of the ith group,

(αβ)ij = interaction of ith group and jth observation date,

eij = error associated with the ith group and jth observation date.

Interventionary studies involving animals or humans, and other studies that require ethical approval, must list the authority that provided approval and the corresponding ethical approval code.

## 3. Results

### 3.1. Vegetation Biomass and Quality, and Hay Quality

**Figure 3:**
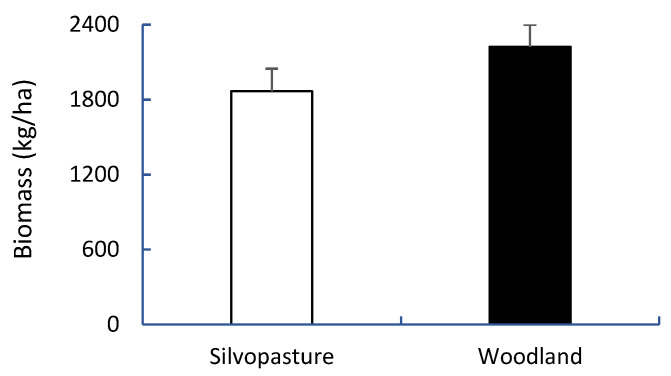
Vegetation biomass (LSMean ± SE) from silvopasture and woodland plots.

**Figure 4:**
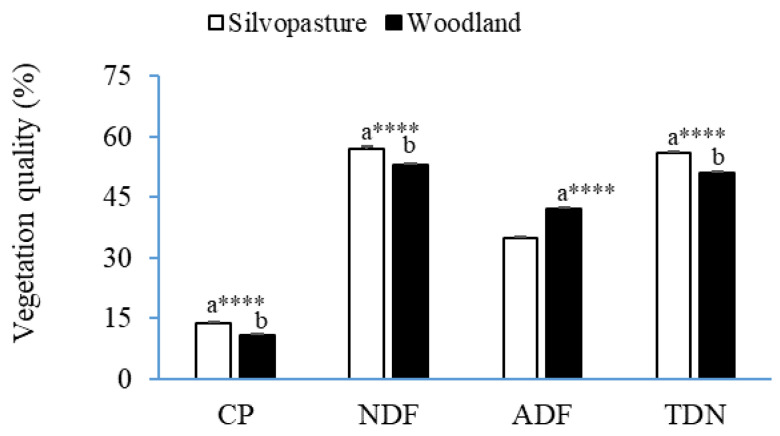
Quality (CP, crude protein; NDF, neutral detergent fiber; ADF, acid detergent fiber; and TDN, total digestible nutrients) (LSMean ± SE) of vegetation from silvopasture and woodland plots. ^ab^ LSMeans within the same variable with different superscripts differ (**** *p* < 0.0001).

There was no difference in biomass productivity between silvopasture and woodland plots (Figure 3). Silvopasture vegetation contained a higher CP (27%), NDF (6%), and TDN (9%) but a lower ADF (15%) than woodland vegetation (*p* < 0.0001) (Figure 4).

The quality of the coastal bermudagrass hay used to supplement animals during the study is presented in Table 3.

### 3.2. Effect of Supplement Type on Animal Performance

The does with supplemental grazing (SG) showed a better FAMACHA score (11%) as compared to the does with supplemental feedstuffs (SF) (Table 4). No difference was found in live weight and body condition scores between the groups of does. The supplement type did not affect the performance (live wt., FAMACHA score, and body condition score) of kids.

Dependent variables in all data sets for animal performance showed significant correlations. The FAMACHA score and live weight were negatively correlated for does. The FAMACHA score and BCS showed a stronger negative correlation for does than the correlation between live weight and FAMACHA score. However, the correlations between live weight and BCS were positive (Table 5).

In kids also, the correlation between the FAMACHA score and BCS was negative, whereas the live weight and BCS were positively correlated (Table 6).

### 3.3. Browsing Height

SG does reached a greater (4%) height to browse vegetation in woodlands vs. SF does (*p* < 0.0001) (Table 7).

### 3.4. Fecal Quality

Overall, the fecal N concentration was greater (18%) in SG does vs. SF does (*p* < 0.0001) (Table 8). The ADF content was higher (14%) in the feces of SG does vs. SF does (*p* < 0.0001) (Table 9). Energy was predicted to be the deficient nutrient in feedstuffs for both groups throughout the study period, except toward the end of the study when protein was found to be the deficient nutrient limiting the performance of SG does (Table 10).

## 4. Discussion

### 4.1. Vegetation Biomass and Quality

The variation in the plant species between the systems (silvopasture vs. woodland) might have resulted in the higher (18%) concentration of ADF in woodland vegetation vs. silvopasture vegetation, as it is well known that different plant species exhibit different levels of nutritional quality [21]. The study of Celaya et al. (2008) [22], who reported a higher CP (115%) and lower ADF (43%) of forages from pastures containing perennial ryegrass (*Lolium perenne* L. cv ‘Phoenix’), hybrid ryegrass (*Lolium* × *hybridum* Hausskn. cv ‘Dalita’), and white clover (*Trifolium repens* L. cv ‘Huia’) vs. woody vegetation of heathland, corroborates the result of the current study. Another reason for the better quality of silvopasture vegetation compared to the woodland vegetation found in the current study might be due to added fertilizers and lime in silvopastures and no such amendments in woodlands, as we were trying to utilize the naturally grown vegetation in woodlands. Under the normal practice, woodlands are not fertilized or limed, unlike in silvopastures, where forages are cultivated following the agronomic practices. Soil fertilization was reported to influence the quality of vegetation [23].

### 4.2. Effect of Supplement Type on Animal Performance

The hypothesis that the lactating does stocked in woodlands would perform equally well with either supplemental grazing or feedstuffs was partially rejected since the FAMACHA score was better in does with supplemental grazing (SG) vs. does with supplemental feedstuffs (SF). The better FAMACHA score in SG does found in the current study could be due to better nutrition obtained from the supplemental grazing vs. the hay and corn supplement. The better nutritional status in SG does might have increased the immunity against H. contortus and resulted in a better FAMACHA score compared to SF does. Because of inadequate nutrients, many body functions, mainly immune functions, are impaired, resulting in a greater establishment, fecundity, and survival of GI parasites within the host [24]. Poor immune response increases the risk of heavy parasitic load and diseases in animals. Animals with adequate nutrition can withstand parasite infestation better than animals on a poor diet [13]. Shrestha et al. (2022) [25] reported a lower incidence of GI parasites in lactating does stocked in woodlands with supplemental grazing vs. supplemental feedstuffs due to better nutrition in the former. The better FAMACHA score of does having access to supplemental grazing in silvopastures when stocked in woodlands in the current study agrees with Tiwari et al. (2021) [26], who found a better FAMACHA score in Kiko does grazing legume-grass vs. sole-grass pastures. Worku et al. (2017) [27] also reported a better FAMACHA score in goats when grazing cowpea pastures vs. pearl millet (*Pennisetum americanum* (L.) Leeke) pastures.

The negative correlation between the FAMACHA score and BCS found in the current study was as expected because the increase in FAMACHA score decreases hematocrit, hemoglobin, and BCS [28]. The correlation between BCS and live weight was positive as expected because the increment in live weight indicates the good nutritional condition of animals with a gain in muscle mass, which leads to the increment in BCS. The results of the negative correlation between BCS and FAMACHA score and positive correlation between BCS and live weight agrees with Karki et al. (2021) [29], who reported similar correlations among performance parameters in meat goats and hair sheep.

### 4.3. Browsing Height

The group of nursing does with supplemental grazing reaching a greater height when browsing vegetation compared to does supplemented with hay and corn observed in the current study could be due to better nutrients, and the former might have been obtained from grazing quality forages in silvopastures. Berhane and Eik (2006) [30] reported higher (9%) browsing activities of Begait and Abergaille goats in rangelands when supplemented with vetch (*Vicia sativa* L.) hay at the rate of 1% of their body weight as compared to no-supplement control. Mkhize et al. (2016) [31] found the increment in browsing time and activity in South African veld goats in rangelands when supplemented with maize (*Zea mays* L.) grain or soybean (*Glycine max* (L.) Merr.) meal at the rate of 100 g per animal as compared to goats without supplement.

### 4.4. Fecal Quality

The hypothesis that the concentration of fecal nutrients would remain similar in nursing does raised in woodlands irrespective of the supplement type was rejected since the concentrations of fecal N and ADF were higher in SG does vs. SF does. The higher concentration of fecal N in SG does vs. SF does in the current study might be due to the higher protein intake by SG does. SG does grazed higher quality foliage (14% CP) as a supplemental grazing in silvopastures, whereas SF does were supplemented with hay (12% CP) and corn (9% CP). The nitrogen in feces is the sum of undigested dietary N, microbial fecal N, and endogenous N. The dietary indigestible N, which contributes to the total fecal N, increases with the amount of protein consumed [32]. Similarly, microbial fecal N, which also contributes to the total fecal N, increases with higher protein intake when adequate energy is available to build microbial cells [32]. Holloway et al. (1981), Holecheck et al. (1982), and Mubanga et al. (1985) [33,34,35] reported a strong positive correlation (r = 0.89) of fecal N and dietary N. Higher fecal N in does with supplemental grazing observed in the current study resembles the finding of Orellana et al. (2020) [17], who reported higher fecal N (71%) in ewes grazing higher CP (29%)-containing vegetation vs. lower CP (16%)-containing vegetation.

Another likely reason for a higher fecal N in SG does as compared to SF does could be due to the higher consumption of condensed tannins present in sericea lespedeza grown in silvopasture plots by SG does. Several studies have reported that condensed tannins bind protein, resulting in the formation of an insoluble tannin–protein complex, which can elevate fecal N concentration irrespective of the dietary N [36,37]. Mould and Robbins (1981) [38] found that condensed tannins in fireweed (*Epilobium angustifolium*) decreased the protein digestibility and increased the fecal N in captive elk. Similarly, Norris et al. (2020) [39] reported higher fecal N (38%) in crossbred steers when Quebracho (*Schinopsis balansae* Engl.) condensed tannin extracts were fed at the rate of 45 g/kg of dietary DM vs. 0 g/kg of dietary DM. The higher fecal ADF found in SG does could be because of their consuming the sericea lespedeza present in a few silvopasture plots used for supplemental grazing. Min and Solaiman (2018) and Pech-Cervantes et al. (2020) [40,41] found that the antimicrobial effects of condensed tannins from sericea lespedeza lowered the community of fibrolytic bacteria, reducing the fiber digestion in sheep and goats. Norris et al. (2020) [39] mentioned that, as the level of condensed tannin increased from 15 g/kg of dry matter to 45 g/kg of dry matter in the diet of crossbred steers, fecal ADF, NDF, DM, and OM were linearly increased (*p* ≤ 0.001). The current study revealed energy as a major deficient nutrient predicted from their fecal nutrients, limiting their performance. This result suggests the need for more energy than that used in the current study to improve the performance of nursing does raised in woodlands. The findings of the current study are applicable to woodlands having similar types of vegetation, climate, plant growth stages, and productive conditions.

## 5. Conclusions

Does with supplemental grazing showed a better FAMACHA score (11%) and browsing ability at a greater height (4%) to consume understory vegetation in woodlands compared to does supplemented with corn (0.5% of metabolic weight) and ad libitum hay. The concentrations of N (18%) and ADF (14%) were greater in the feces of does with supplemental grazing vs. does with supplemental feedstuffs. The findings of this study demonstrated that grazing quality pastures is a better option than supplementing with the feedstuffs used in the current study for improving the performance (FAMACHA score) and browsing activities of nursing does raised in woodlands. Further study is recommended to evaluate the effect of condensed tannins on the nutrient dynamics of Kiko goats.

## Figures and Tables

**Table 1:** List of major understory plant species in woodland plots.

S.N.	Common Name	Scientific Name
1	American beautyberry	*Callicarpa americana* L.
2	Blackberry	*Rubus* L.
3	Broomsedge	*Andropogon* L.
4	Dogfennel	*Eupatorium capillifolium* Lam.
5	Grapevine	*Vitis rotundifolia* Michx.
6	Greenbrier	*Smilax* L.
7	Hickory	*Carya* spp. Nutt.
8	Panicgrass	*Panicum* L.
9	Persimmon	*Diospyros virginiana* L.
10	Southern red oak	*Quercus falcate* Michx.
11	Sumac	*Rhus* spp. L.
12	Sweetgum	*Liquidambar* L.
13	Water oak	*Quercus nigra* L.
14	Wild plum	*Prunus americana* Marshall
15	Winged elm	*Ulmus alata* Michx.
16	Yaupon	*Ilex vomitoria* Aiton.

**Table 2:** Initial parameters of does and kids used in the woodland study.

Variables	SG		SF	
	Does (9)	Kids (18)	Does (8)	Kids (15)
	LSMean ± SE			
Live wt. (kg)	42.1 ± 2.13	11.9 ± 0.47	42.0 ± 2.18	12.2 ± 0.87
FAMACHA score	2.6 ± 0.24	2.9 ± 0.16	2.6 ± 0.26	2.7 ± 0.19
BCS	2.3 ± 0.08	2.9 ± 0.08	2.3 ± 0.09	3.0 ± 0.09

**Table 3:** Quality of coastal bermudagrass hay used to supplement goats in woodland plots.

Quality Parameter	Coastal Bermudagrass Hay
	% (LSMean ± SE)
Crude protein	12 ± 0.1
Neutral detergent fiber	71 ± 0.2
Acid detergent fiber	30 ± 0.2
Total digestible nutrient	67 ± 0.2

**Table 4:** Live weight, body condition score (BCS), and FAMACHA score of does with supplemental grazing (SG) or feedstuffs (SF) while stocked in woodlands.

Observation Date	Live Weight (kg.)		BCS		FAMACHA Score	
	SG	SF	SG	SF	SG	SF
	LSMean ± SE					
16 July	39.8 ± 1.76	41.5 ± 1.87	2.3 ± 0.1	2.4 ± 0.11	2.8 ± 0.22	2.8 ± 0.23
30 July	40.2 ± 1.76	42.4 ± 1.87	2.0 ± 0.1	1.9 ± 0.11	2.4 ± 0.22	2.6 ± 0.23
13 August	39.6 ± 1.76	39.6 ± 1.87	2.2 ± 0.1	2.1 ± 0.11	2.9 ± 0.22	3.3 ± 0.23
27 August	38.2 ± 1.76	40.2 ± 1.87	2.1 ± 0.1	2.3 ± 0.11	2.6 ± 0.22	3.0 ± 0.23
10 September	39.8 ± 1.76	40.4 ± 1.87	2.2 ± 0.1	2.3 ± 0.11	2.2 ± 0.22 ^b^	2.9 ± 0.23 ^a^*
24 September	39.6 ± 1.76	39.7 ± 1.87	2.4 ± 0.1	2.4 ± 0.11	2.6 ± 0.22	2.6 ± 0.23
8 October	40.6 ± 1.76	42.0 ± 1.87	2.5 ± 0.1	2.4 ± 0.11	2.1 ± 0.22	2.3 ± 0.23

^ab^ LSMeans in a row within the same variable with different superscripts differ (* *p <* 0.05).

**Table 5:** Correlation among live weight, body condition score (BCS), and FAMACHA score of does in woodlands.

	Both Group		SG		SF	
	FAMACHA Score	BCS	FAMACHA Score	BCS	FAMACHA Score	BCS
Live weight	−0.22 *	0.48 ****	−0.24 *	0.42 ***	−0.23	0.40 ***
FAMACHA score		−0.43 ****		−0.42 ***		−0.38 **

* *p* < 0.05, ** *p* < 0.01, *** *p* < 0.001, **** *p* < 0.0001.

**Table 6:** Correlation among live weight, body condition score (BCS), and FAMACHA score of kids in woodlands.

	Both Group		SG		SF	
	FAMACHA Score	BCS	FAMACHA Score	BCS	FAMACHA Score	BCS
Live weight	−0.49 ****	0.52 ****	−0.47 ****	0.39 ****	−0.52 ****	0.59 ****
FAMACHA score		−0.39 ****		−0.33 ****		−0.43 ****

**** *p* < 0.0001.

**Table 7:** Browsing height of does with supplemental grazing (SG) or feedstuffs (SF) in woodlands.

	SG	SF
	**m (LSMean ± SE)**	
Browsing height	1.6 ± 0.01 ^a^****	1.5± 0.01 ^b^

^ab^ LSMeans in the same row with different superscripts differ (**** *p* < 0.0001).

**Table 8:** The concentration of nitrogen and phosphorus in the feces of does with supplemental grazing (SG) or supplemental feedstuffs (SF) in woodlands.

Observation Date	Fecal Nitrogen		Fecal Phosphorus	
	SG	SF	SG	SF
	% (LSMean ± SE)			
12 July	2.1 ± 0.08	2.2 ± 0.08	0.7 ± 0.03	0.7 ± 0.03
13 August	2.4 ± 0.08 ^a^***	2.0 ± 0.08 ^b^	0.6 ± 0.03 ^a^*	0.5 ± 0.03 ^b^
9 September	2.3 ± 0.08 ^a^****	1.8 ± 0.08 ^b^	0.6 ± 0.03	0.5 ± 0.03
7 October	2.2 ± 0.08 ^a^****	1.6 ± 0.08 ^b^	0.4 ± 0.03	0.4 ± 0.03

^ab^ LSMeans in a row under the same variable with different superscripts differ (* *p* < 0.05, *** *p* < 0.001, **** *p* < 0.0001).

**Table 9:** Fecal quality (NDF, ADF, and TDN) of does with supplemental grazing (SG) or supplemental feedstuffs (SF) in woodlands.

Observation Date	NDF ^†^		ADF		TDN	
	SG	SF	SG	SF	SG	SF
	% (LSMean ± SE)					
12 July	58.0 ± 0.55	59.1 ± 0.58	39.1 ± 0.63	40.5 ± 0.67	58.5 ± 0.32	57.9 ± 0.34
30 July	58.8 ± 0.55	57.8 ± 0.58	47.6 ± 0.63 ^a^****	38.3 ± 0.67 ^b^	58.0 ± 0.32	58.7 ± 0.34
13 August	63.5 ± 0.67	62.9 ± 0.50	51.2 ± 0.77 ^a^**	47.8 ± 0.57 ^b^	55.3 ± 0.39	55.7 ± 0.29
27 August	65.5 ± 0.55	66.5 ± 0.58	54.5 ± 0.63 ^a^****	45.3 ± 0.67 ^b^	54.1 ± 0.32	53.6 ± 0.34
10 September	62.5 ± 0.55 ^a^*	60.9 ± 0.58 ^b^	50.4 ± 0.63 ^a^****	42.4 ± 0.67 ^b^	55.9 ± 0.32 ^b^	56.9 ± 0.34 ^a^*
24 September	65.5 ± 0.55	65.9 ± 0.58	50.4 ± 0.63 ^a^**	47.7 ± 0.67 ^b^	54.1 ± 0.32	53.9 ± 0.34
8 October	60.7 ± 0.55 ^a^*	59.0 ± 0.58 ^b^	49.3 ± 0.63 ^a^****	37.5 ± 0.67 ^b^	57.0 ± 0.32 ^b^	58.0 ± 0.34 ^a^*

^ab^ LSMeans in a row under the same variable with different superscripts differ (* *p* < 0.05, ** *p* < 0.01, **** *p* < 0.0001); ^†^ NDF—Neutral detergent fiber; ADF—Acid detergent fiber; TDN—Total digestible nutrients.

**Table 10:** The major nutrient limiting the performance of does with supplemental grazing or feedstuffs while stocked in woodlands.

Observation Date	Limited Nutrient	
	SG	SF
12 July	Energy	Energy
13 August	Energy	Energy
9 September	Energy	Energy
8 October	Protein	Energy

## Data Availability

Data supporting the findings of this study are available to anyone from the corresponding author upon reasonable request.
